# Stem cell models as an *in vitro* model for predictive toxicology

**DOI:** 10.1042/BCJ20170780

**Published:** 2019-04-15

**Authors:** Stephen Lynch, Chris S. Pridgeon, Carrie A. Duckworth, Parveen Sharma, B. Kevin Park, Chris E.P. Goldring

**Affiliations:** Department of Molecular and Clinical Pharmacology, MRC Centre for Drug Safety Science, University of Liverpool, Sherrington Building, Ashton Street, Liverpool L69 3GE, U.K.

**Keywords:** cardiotoxicity, gastrointestinal toxicity, hepatotoxicity, organoid, stem cell, toxicology

## Abstract

Adverse drug reactions (ADRs) are the unintended side effects of drugs. They are categorised as either predictable or unpredictable drug-induced injury and may be exhibited after a single or prolonged exposure to one or multiple compounds. Historically, toxicology studies rely heavily on animal models to understand and characterise the toxicity of novel compounds. However, animal models are imperfect proxies for human toxicity and there have been several high-profile cases of failure of animal models to predict human toxicity e.g. fialuridine, TGN1412 which highlight the need for improved predictive models of human toxicity. As a result, stem cell-derived models are under investigation as potential models for toxicity during early stages of drug development. Stem cells retain the genotype of the individual from which they were derived, offering the opportunity to model the reproducibility of rare phenotypes *in vitro*. Differentiated 2D stem cell cultures have been investigated as models of hepato- and cardiotoxicity. However, insufficient maturity, particularly in the case of hepatocyte-like cells, means that their widespread use is not currently a feasible method to tackle the complex issues of off-target and often unpredictable toxicity of novel compounds. This review discusses the current state of the art for modelling clinically relevant toxicities, e.g. cardio- and hepatotoxicity, alongside the emerging need for modelling gastrointestinal toxicity and seeks to address whether stem cell technologies are a potential solution to increase the accuracy of ADR predictivity in humans.

## Introduction

Adverse drug reactions (ADRs) are the harmful side effects of medicine which may occur after acute or chronic exposure to a compound. They place a significant financial burden on the healthcare industry and contribute to ∼5% of all hospital admissions [1]. Similarly, ADRs are burdensome to pharma, who face financial loss due to drug withdrawal if toxicity is detected post-marketing. The current cost of developing a drug is $648 million USD over 10–15 years [2], which is a major concern given that many drugs do not exhibit ADRs until late-stage trials when large investments of time and money have already been made. Between 1980 and 2009, 15% of licenced drugs having proven efficacious in phase II trials were terminated with the main reasons being unanticipated cardiotoxicity, hepatotoxicity and gastrointestinal (GI) toxicity [3]. Currently, cardiac drug-induced injury is the leading cause of drug withdrawal, whilst drug-induced liver injury is the second leading cause of drug termination and causes ∼50% of acute liver failure [4]. Unpredicted cardiotoxicity is the main reason for drug termination, accounting for 28% of drug withdrawals [5]. In the U.S.A., the GI tract has been associated with 20% of drug-induced events [6].

Human embryonic stem cells (hESCs) were initially discovered in blastocyst stage embryos, whereas induced pluripotent stem cells (iPSCs) were developed approximately 13 years ago, by reprogramming adult somatic cells by overexpression of the Yamanaka factors: Oct4, Sox2, Klf4 and cMyc (OSKM) [7]. Both hESCs and iPSCs are pluripotent, meaning that they can differentiate into any somatic cell type. IPSCs can be derived from individuals known to have unusual or diseased phenotypes, these phenotypes can then be introduced into *in vitro* assays at early stages during toxicity testing. Genetic modification platforms such as CRISPR-Cas9 can be utilised to generate isogenic human iPSC lines that can be used to assess cross-organ ADR responses from the same patient. The process for deriving iPSCs is minimally invasive requiring only a skin biopsy or blood sample, though any tissue type can be used.

Stem cells are able to be exploited in both 2D and 3D, therefore, utilising spheroids and organoids to model *in vivo* systems. Spheroids are self-aggregating clusters of cells that form spontaneously when adherent cells are denied an attachment surface. Spheroids have been observed for almost as long as cell culture has been practiced with observations of cells ‘rounding off into little spheres’ as early as 1907 [8]. Due to their 3D physiology, spheroids can be used to replicate a 3D microenvironment which is thought to improve the relevance of cell models to organ parenchyma. Despite their advantages over monolayer systems, spheroids are not perfect as they are typically hypoxic above a given size due to the diffusion gradient of oxygen through non-vascularised tissue, which often leads to necrosis in the core. Despite hypoxia being observed in spheroids, most 2D and 3D cultures are said to be in hyperoxic conditions (21% O_2_), which causes hepatocyte de-differentiation and therefore a loss of specific cell functions. Notably, 5% O_2_ is very effective in maintaining epithelial morphology and retain hepatic functions for up to 5 days [9]. Due to their necrotic core, spheroids can be alternatively used to model small tumour growth, as angiogenesis has not yet occurred *in vivo* and accurately models an oxygen gradient in a tumour microenvironment. Generally, spheroids are comprised of a single cell/tumour cell type. Whilst spheroids are useful in modelling 3D culture, due to their necrotic core and lack of vasculature, spheroids can be used to model tumour growth. Whilst *in vitro* cultures are not entirely representative of normal tissue, mixed populations of non-transformed cells are possible, as is layering with multiple cell types to recapitulate organ structure. Crucially, spheroids do not spontaneously form organotypic structures or differentiate into more mature tissues.

Organoids are a 3D heterogeneous collection of cells, self-organising to recreate organ microanatomy. Organoids self-arrange with a central lumen, allowing for more complete drug penetration and the establishment of a toxicological gradient. Organoids are often derived from tissue-resident stem cells, thus making biopsy of the target tissue necessary. This has several disadvantages compared with iPSC-derived models, which can be derived from easily accessible cells [10], including collection time, patient consent and the volume of culture material. However, organoids remain a promising nascent model for examining toxicity in multiple tissue types. Moreover, some studies have shown that organoids may be produced from iPSCs and exhibit the phenotype of the patient overcoming this limitation of organoid culture [11]. A study conducted by the Clevers group has demonstrated that hepatic organoids express a similar level of CYP3A4 compared with freshly isolated hepatocytes and was verified via midazolam metabolism. This study highlighted the potential of hepatic organoids, replicating a clinical level of metabolism, whilst adopting key 3D functions to mimic an *in vivo* system [12].

## Hepatotoxicity

The liver is the major site of metabolism and detoxification of xenobiotic compounds, which leads to high incidences of ADRs, therefore, hepatoxicity is a leading cause of attrition in drug development. Human primary hepatocytes (hPH) are considered the gold standard for studying *in vitro* hepatotoxicity though they are not a flawless model. A major limitation of using mature hPH is that they rapidly de-differentiate immediately after the tissue is removed from the patient's blood supply and continue to de-differentiate over approximately 1-week post-isolation in culture [13]. Moreover, de-differentiation causes a large reduction in expression of key hepatic proteins, e.g. cytochromes P450 and other key phase I/II enzymes and transporters, which directly affect drug metabolism and therefore the validity of the cell model.

To overcome the limitations of hPH stability *in vitro*, a more physiologically relevant and stable model is required to measure the long-term effects of drug-induced toxicity. Immortalised cell lines, such as HepG2 and HepaRG cells can be used, primarily due to their highly proliferative nature and ease of culture. However, these cell lines are far less physiologically relevant compared with hPHs often lacking many key hepatic proteins. iPSC-derived hepatocyte-like cells (iPSC-HLCs) are a potential ‘Goldilocks’ model combining the replicative nature of cell lines with the potential for close physiological relevance offered by primary cells. They are also able to maintain the phenotype of a single donor allowing repeat experiments under similar genetic conditions, something that is typically not possible with primary cells. Despite their promise, iPSC-HLCs do not currently fully recapitulate the phenotype of freshly isolated hPHs, and, like hPH, do not proliferate when differentiated. However, only a few phenotypic markers are used, which suggests an inadequate benchmark for hepatocyte phenotyping [3].

The phenotype of hepatic models can be examined using drugs previously known to be metabolised by specific enzymes. Several key drugs were analysed, namely, phenacetin (CYP1A2), diclofenac (CYP2C9), omeprazole (CYP2C19), metoprolol (CYP2D6) and midazolam (CYP3A4) [14]. HepaRG and iPSC-HLCs displayed similar metabolic rates after 8 days, except in the case of CYP3A4-dependent metabolism, where iPSC-HLCs showed substantially higher metabolism (Table 1). The iPSC-HLCs demonstrated stable metabolic rates of all tested cytochromes P450 until day 29, except for CYP3A4 which showed a sharp decrease to levels similar to those observed in HepaRG cells at day 9 (Table 1).

**Table 1:** Enzyme metabolic rates, depending on the concentration of product produced, comparing immortalised HepaRG cells against iPSC-Hep cell lines [14], showing the different systems responding to a known compound over 8 and 29 days. IPSCs responded in a far more stable manner over 29 days, as HepaRG cells metabolic rates were too low to measure

Cell model	Day	Cell per well	Drug oxidation activity pmol products/hour/1 × 10^5^ cells				
			CYP1A2	CYP2C9	CYP2C19	CYP2D6	CYP3A4
HepaRG	8	1.8	0.93 ± 0.03	0.040 ± 0.004	4.1 ± 1.0	11 ± 2.0	20 ± 2.0
iPSC #1	8	1.5	0.93 ± 0.24	0.004 ± 0.001	1.2 ± 0.20	7.0 ± 2.0	94 ± 20.0
	29	1.1	1.2 ± 0.10	0.053 ± 0.006	7.9 ± 0.80	24 ± 4.0	26 ± 2.0
iPSC #2	8	1.5	0.78 ± 0.19	0.002 ± 0.0004	1.0 ± 0.10	4.4 ± 1.1	77 ± 6.0
	29	1.3	1.2 ± 0.10	0.027 ± 0.005	6.5 ± 0.70	16 ± 1.0	24 ± 3.0

IPSC-HLCs have shown early success in safety assessments, particularly with regard to their ability to reproducibly model diseased phenotypes. High-throughput screening is widely applied in drug development to prioritise lead molecules, to decrease animal use [15], allowing for Tox21/ToxCast programmes to aid decision-making [16] during drug discovery. The necessity to generate data on the potential toxicity of at least 30,000 compounds, is expected to require up to 10 million animals [17]. In one study, iPSCs were derived from patients with α1antitrypsin (A1AT) deficiency [18], and through high-throughput screening, a compound that significantly reduced defective A1AT within the cytoplasm was identified [19]. As a result, iPSCs from diseased donors are being investigated for other conditions, including hypercholesterolaemia [20], glycogen storage disease [20–23], Gaucher's disease [24], hereditary tyrosinaemia [20], hereditary cholestasis [18] and defective mitochondrial respiratory chain complex disorder [23].

Human skin-derived precursors (hSKP) are a multipotent stem cell line that can be differentiated towards a hepatic fate (hSKP-HPC). The response of hSKP-HPC to paracetamol (APAP) was recently examined using qPCR analysis [25]. Notably, CYP3A5, a foetal isoform of CYP3A4, demonstrated a 15-fold increase in hSKP-HPC upon APAP exposure demonstrating the capacity for induction of key metabolic enzymes in these cells [20]. Basal levels of several CYPs in hSKP-HPC have been found to be minimal [26], however, it has been hypothesised that the CYP expression may fluctuate depending on the xenobiotic to which they are exposed.

Like most cells, stem cells are currently most commonly cultured in 2D conditions. This is a major limitation since these models cannot completely mimic the physiology of an *in vivo* system e.g. no connective tissue or the presence of extracellular matrix. 2D systems have contributed to a poor standard of pre-clinical *in vitro* hepatotoxicity assays. Consequently, more than 90% of drugs that yield positive data during *in vitro* pre-clinical studies fail the safety margins required in subsequent clinical trials. As a result of a lack of key phenotypic properties in 2D models, 3D models are currently being explored to improve the reliability of *in vitro* toxicity assays [26].

HepG2 cells in a typical rigid 3D culture show improved hepatic functions over monolayer cultures in terms of glycogen storage, bile salt transportation, development of bile canaliculi and increased expression of CYP3A4, CYP2E1, CYP2C9, CYP2C19, CYP2D6 and UGT1A1 [26]. Drug-induced cholestasis (DIC) is measured *in vitro* by the compound's ability to inhibit bile salt export pump (BSEP), however, DIC is typically complex, multifactorial and delayed in its manifestation [27–29], and therefore innovative toxicity models are required. Spheroid models formed via hPH and HepaRG cells were dosed with cholestatic-inducing compound chlorpromazine [30]. Both models displayed DIC related toxicity, whilst the HepaRG spheroid also displayed reduced F-actin expression, indicating chlorpromazine in disrupting structural integrity, which has been implicated in early chlorpromazine-induced cholestasis through oxidative stress [31].

Organoid models are being developed in the hope that they will improve upon current 3D models. When compared with human foetal liver progenitor cell-derived hepatocytes (hFLPC-HLCs) human liver organoids showed far greater urea and albumin production. After 21 days human liver organoids produced ∼4.4-fold higher albumin concentrations and 4.0-fold higher urea concentrations than hFLPC-HLCs. These results show that organoids are potentially capable of greater physiological relevance than traditional 2D culture systems [32]. It has been suggested that isolated primary hepatocytes can be used to generate branched hepatocyte-like organoids (Hep-Org), when exposed to a variety of small molecules, including RSPO-1 and other Wnt agonists [33]. Through biliary ductal cell isolation, it is possible to generate cholangiocyte-like organoids (Chol-Org), which differ greatly from Hep-Org, as shown through RNA analysis, and through lineage tracing, loosely indicates a similarity between primary hepatocytes and Hep-Org, however, Hep-Orgs have shown decreased HNF4a and albumin expression, 2- and 4-fold decrease, respectively, whilst Chol-Orgs demonstrated >1000-fold decrease [33]. Notably, genes involved in hepatocyte functions such as cytochrome P450 activity, glycogen metabolism, lipid metabolism, steroid metabolism, urea cycle and complement activation all displayed very similar expression profiles between Hep-Orgs and primary hepatocytes [33].

## Cardiotoxicity

IPSC-derived cardiomyocytes (iPSC-CMs) have great potential in cardiotoxicity research. They offer an attractive platform to mimic cardiovascular diseases, model early stages of cardiac development and advance predictive toxicology assays *in vitro*. Like other iPSC-derived models, iPSC-CMs retain the donor phenotype potentially allowing multiple experiments on rare phenotypes and have been used to model complex channelopathies such as prolonged QT syndrome, catecholaminergic polymorphic ventricular tachycardia and familial hypertrophic cardiomyopathy (HCM) [34–36], alongside those seen in Table 2. The predictive capability of iPSC-CMs is therefore dependent on key structural characteristics and electrophysiology *in vitro*.

**Table 2:** Cardiac diseases which have been modelled *in vitro* using iPSC-CMs. Many drugs have been tested using disease state iPSC-CMs

Disease	Tested drug(s)
LQT1	Propranolol [77], isoprenaline [78]
LQT2	Nifedipine [38], propranolol [79], allele-specific siRNA [80], cisapride [81]
LQT8	Roscovitine [39]
CPTV1	Isoprenaline, forskolin [35]
DCM	Metoprolol, norepinephrine [82]
HCM	Propranolol, verapamil, nifedipine [36]
ARVC	Nifedipine [83], isoproterenol [84]

A key benefit to using iPSC-CMs is phenotypic retention, which is the ability to model drug-induced cardiotoxicity in diseased states. Cardiomyocytes were dosed with 1–300 nM of cisapride, a potent HERG channel blocker, using control human embryonic stem cell-derived cardiomyocytes (hESC-CMs) and hESC-CMs from long QT syndrome (LQT), HCM and dilated cardiomyopathy (DCM) patient-derived cardiomyocytes (Table 2). Cisapride was observed to provoke early after depolarisation (EAD) at different concentrations for each diseased cell type [37]. Nicorandil, a K^+^ channel opener, was next applied to elicit drug-induced responses in disease cell models. Nicorandil has been implicated in shortening the QT interval through increased K^+^ efflux, therefore inducing arrhythmias [38–40]. Upon Nicorandil application to diseased cardiomyocytes, 100 nM was found to both normalise APD prolongation and remove spontaneously occurring EADs. Many studies also reported arrhythmia induction in patients receiving high levels of Nicorandil due to excessive QT shortening [41–43]. Several key disease phenotypes have also been modelled (Table 2), with successful toxicology studies performed.

In a recent study, hESC-CMs were exposed to a range of cadmium chloride (CdCl_2_) concentrations to induce morphological changes. Consistent with previous studies, significantly higher levels of reactive oxygen species (ROS) were observed in CdCl_2_ treated cells compared with control [44–46]. In addition, hESC-CMs displayed sarcomeric disorganisation and disruption, increased nucleoplasmic ratio and nuclear membrane shrinkage [47]. These results are in concordance with *in vivo* studies, in which CdCl_2_ disrupts cardiac structure and integrity. These results show that hESC-CMs can recapitulate complex cardiac structure and electrophysiology, which is important for accurate prediction of cardiotoxicity.

A critical function of cardiomyocytes is their contractile ability, therefore, the effects of E-4031 (a class III antiarrhythmic compound) was considered to examine antiarrhythmic effects. Administration of E-4031 at 30–100 nM, resulted in a significant decrease in beating rate (32.7 ± 1.2/min) versus vehicle control and a decrease in contractile velocity versus vehicle control [48]. The recent work of the Wu group is an important advance as it shows that even for a complex, off-target toxicity such as doxorubicin-induced cardiotoxicity, which likely entails decreased mitochondrial function, perturbed calcium regulation and oxidative stress, it is possible to emulate this using patient-derived single cell iPSC-CMs [49]. A critical function of cardiomyocytes is their contractile ability, therefore, the antiarrhythmic effects of E-4031(a class III antiarrhythmic compound) were examined. Administration of E-4031 significantly decreased contractile velocity and beating rate in iPSC-CTs (32.7 ± 1.2/min) [48]. Dose-dependent doxorubicin toxicity has been successfully modelled in iPSC-CTs, reporting the reported minimum effective concentration of doxorubicin in monolayer iPSC-CM systems is not significantly different from the concentrations achieved in pre-clinical studies, therefore, indicating a comparable tolerance to cytotoxins between systems [48,50–52].

A recent study treated cardiomyocyte spheroids with antibiotic, antidiabetic and anti-cancer drugs [52]. The spheroids physiologically showed relevant structure and metabolic functions, as 6/8 known cardiotoxic compounds tested were also detected *in vitro*, therefore, CM spheroids correctly identified 75% of cardiotoxic compounds, compared with clinical data. The study has found 3D cardiac tissue models tested in this manner are more sensitive to cardiotoxicity than traditional viability assays. Another recent study has reported that rosiglitazone; a potent antidiabetic drug, induced severe contractile failures in mice at 10–30 µM [53], when iPSC-CM-derived spheroids were treated in a similar manner, the microtissue stopped contracting at 50 µM. This study suggests that spheroids are not currently as sensitive as an *in vivo* model, and whilst they are not a perfect model, they can exhibit physiological changes in response to an insult. Spheroids exhibit action potential propagation, force transduction and contractile tension, which play a critical role in pharmacologically induced responses of cardiomyocytes and show increased expression of cardiac troponin (cTnT) and aMHC [54–56].

There are, however, shortcomings with iPSC-CMs which must be overcome to establish a reliable *in vitro* model. The maturity of iPSC-CMs must be improved to establish predictive electrical, mechanical and metabolic function. Stem cell differentiation protocols can lead to variability, depending on the protocol used. This can lead to differences in phenotype and variation in the proportion of cell types produced, e.g. atrial-like cells or pacemaker-like cells. A common problem with 3D cardiomyocytes is the extracellular matrix affecting the contraction and relaxation of the system which may restrict the ability to contract due to the attachment to a rigid matrix. To negate the effect of a matrix upon 3D-CMs, it is possible to generate ‘free-floating’ spheroid like structures, e.g. hanging droplet formation, which displays beating cardiomyocytes.

## Gastrointestinal toxicity

The GI tract plays an important role in xenobiotic bioactivation, metabolism and detoxification, as it is enriched with xenobiotic processing proteins. The epithelium of the GI tract has a rapid cell turnover, involving the proliferation of stem cells at the base of the crypt which produces daughter cells that then migrate along the crypt–villus axis. Intestinal stem cells either divide asymmetrically, which leads to one identical daughter stem cell and one transit amplifying cell that initially retains some proliferative capacity, but eventually becomes a committed progenitor. Rarely, stem cells divide symmetrically giving rise to two stem cells. The Wnt target gene leucine-rich-repeat-containing G-protein-coupled receptor 5 (LGR5) [57] is expressed in cells found at the base of a crypt and is widely believed to label active cycling stem cells. Lineage tracing using LGR5 has shown that all five differentiated intestinal epithelial cells arise from these stem cells: columnar epithelium (mainly absorptive enterocytes), goblet cells, Paneth cells, tuft cells and neuroendocrine cells [58]. LGR5 positive cells are located at the base of small intestinal and colonic crypts in crypt base columnar cells, which are thought to be active adult intestinal stem cells [11] and self-renew with an ∼24-h cell cycle time [59]. Therefore, LGR5 detection would allow for efficient characterisation of adult stem cells *in vitro*. Whilst adult stem cells are source-limited in comparison with iPSC enteroids, they enable spontaneous formation of organoid structures, maintaining any genetic mutations to model disease states. Also, tissue-derived organoids can be generated more cheaply and with greater efficiency than iPSC enteroids, since differentiation is not required.

GI organoids are well established, characterised and have a relatable metabolic profile compared with donor matched tissue [58]. GI organoids are generated via stem cell isolation from the intestinal crypt [60]. Crypt cultures form spherical structures almost immediately, with small buds appearing typically after 5 days [11]. Human-derived GI organoids require a culture in differentiation medium to generate all mature cell lineages [61] and there are subtle differences in growth medium and dynamics between small intestinal-derived enteroids and colon-derived colonoids [62]. Multiple phase I/II drug-metabolising enzymes and transporters have been identified in enteroids [10]. Stable crypt cultures express high levels of CES1/2, UTG1A1 and key drug transporters ABCC2 and ABCB1A [63].

Irinotecan is a potent anti-cancer drug, which upon hydrolysation by carboxylesterases (CES) forms the active topoisomerase inhibitor SN-38, which undergoes glucuronidation by UGT1A1 to form SN-38G. Cytochrome P450 3A4 (CYP3A4), is one of the most versatile drug-metabolising enzymes, accounting for the metabolism of approximately half of all prescribed medications [64] and is responsible for hepatic metabolism of irinotecan. Typically, the bioactivation and detoxification of SN-38 have been accredited to hepatic pathways [63] through CYP3A4 metabolism, though CYP3A4 is also present in the GI tract. However, recent studies using GI organoids have implicated intestinal CES2 with a greater affinity for irinotecan metabolism than hepatic CES1 [59]. GI organoid mediated irinotecan metabolism can be measured via intra-/extracellular concentrations of irinotecan/SN-38/SN-38G and can be used to quantify bioactivation and detoxification using HPLC-MS analysis [65]. Further metabolic studies have shown that the presence of metabolites was detected in the extracellular medium, rising in concordance with irinotecan concentration. Intracellular SN-38 was significantly higher than SN-38G, which suggests significant efflux of SN-38G. GI organoids derived from UGT1A1 knockdown mice retained their genotype and did not express UGT1A1, as a result, SN-38G was not detected [65].

Intestinal organoids can replicate typical transport physiology, alongside host–pathogen interactions. Therefore, human intestinal organoids (HIO) can be employed as a model to understand salt/water transport and diarrhoea-related pathophysiology. Human GI organoids have been shown to express sodium and chloride transporters (NHE3 and DRA respectively), chloride efflux channels (CFTR and BLM NCKCC1), sodium/potassium-ATPase and potassium channels [66].

GI organoids are a useful model to create disease states *in vitro*, allowing for genetic and pathogenic influences to be analysed. Activation of cAMP signalling increases the membrane potential in wild type but not CFTR^−/−^ murine organoids [67], upon treatment with forskolin, apical fluid secretion is observed, leading to intestinal cell shrinkage and organoid expansion allowing for a useful model to monitor intestinal CFTR-related fluid secretion [67,68], which has enabled the development of a pre-clinical screening assay to assess how patients respond to expensive cystic fibrosis drugs [69]. Diarrhoea, cholera, rotavirus and enterohaemorrhagic *Escherichia coli* (EHEC) are amongst the major causes of death worldwide [70]. Both cholera and rotavirus inhibit NHE3, by causing the GPCR to remain in an active state, leading to an increase in intracellular cyclic AMP which continually activates the CFTR [71]. Previous studies have also used iPSC-derived organoids in studying rotaviral infection [72]. HIO treated with a calcium/calmodulin kinase 2 inhibitor (STO-609) have been found to have significantly lower rotavirus infection, quantified by infectious virus particle production [66]. EHEC infection results in GI damage leading to micropinocytosis induction [72] which is indicative of symptoms found *in vivo*. IPSC-derived HIO exposed to a known antigen known to induce an identical response to EHEC, undergo large cytoskeletal changes, resembling micropinocytosis, highlighting the utility of GI organoids as an *in vitro* model [67].

Human colonic organoids, isolated from healthy and adenocarcinoma tissue have been used to screen compounds, to establish their effect on patients with different genomes. Adenocarcinomic organoids derived from patients that exhibited a loss-of-function mutation in TP53 showed resistance to MDM2 inhibitor nutlin-3a, whilst ‘healthy’ organoids showed cell death. This has been further demonstrated when organoids from colonic tumours expressing KRAS mutations, exhibited a resistance to cetuximab (anti-EGFR inhibitor) [73]. Through genome sequence analysis, performed on organoids derived from a patient diagnosed with gastric cancer and metastasis, the presence of TFGBR2 was identified as a genetic factor for increased risk of metastasis [74]. Utilising CRISPR-Cas9, colorectal organoids can be effective in pre-clinical drug screening assays. In a KRAS wild-type colon tumouroid cell line that was sensitive to combinational therapy (EGFR and MEK inhibitors), the introduction of a KRAS^G12D^ mutation via CRISPR resulted in a loss of drug sensitivity. As the genetic makeup of these isogenic lines can be said to be identical before genome editing, this finding proved that it was indeed the RAS status of the tumour that was responsible for the loss of drug sensitivity. Therefore, CRISPR-guided genetic modification of organoids can serve to strengthen conclusions based on large drug screens [75], however, this technology is best served once the mechanism behind the disease is known. Modified isogenic cells provide little information in detecting ADRs that were not detected in humans during pre-clinical testing. Isogenic cells can, however, provide a very useful resource upon confirming a genetic disposition towards ADRs.

## Conclusion

Patient-derived iPSC/hESCs can play a role in drug discovery and pre-clinical toxicity testing for the treatment of patients with GI, cardiovascular and liver diseases. Current drug testing platforms, such as animal studies and human clinical trials are not comprehensive, therefore, human iPSCs may provide an advantage which can enhance the current approaches to drug toxicology studies. Moreover, the ability of human iPSCs to simulate organ systems *in vitro* may allow studying the effects of drug metabolites on various cell types. Many studies have utilised iPSC-based systems, in both 2D and 3D, to identify potential ADRs through disease modelling, metabolism studies and proteomic studies. Due to the phenotypic retention of iPSCs, the efficiency of differentiation and epigenetic shadowing, disease states can be modelled, allowing for patient-specific *in vitro* analysis. Whilst the application of iPSCs in toxicity testing and drug development represents a very advanced and practical use of stem cells, the acceptance and incorporation of the pharmaceutical industry to implement a new approach to current methods will be a slow process [76]. Whilst iPSC-derived hepatocytes are a valuable resource, they are not yet considered to be a ‘perfect’ mature hepatocyte, which may limit their use *in vitro*, despite their many advantages: cost-effectiveness, an unlimited source of hepatocytes, and the potential of high-throughput screening.

Organoids are another promising model for *in vitro* toxicity, currently there are several issues preventing their widespread use. Organoids are fragile in culture since they rely on a gel-like scaffold of BME-2 or Matrigel to grow, limiting their scale-up efforts. Another concern is that the organoid culture methodology is currently under patent, which may potentially limit their availability to be used commercially. With the current technology available, it is unlikely iPSC/hESC-derived systems and patient-derived organoids are ready to replace animal models, this is mainly due to the complexity of recreating an *in vivo* system where every tissue is represented. Organoids cannot recreate a fully vascularised system and incorporate multiple cell types, e.g. macrophages and fibroblasts. In the future, we hope the field can advance in such a manner that allows for the co-culture of organoids with other cell types whilst retaining a physiologically relevant structure. The flaws listed however are synonymous amongst most *in vitro* systems and not exclusive to stem cell systems. In conclusion, no current system can be said to be perfect, but due to the advantages and flexibility of stem cell systems, in both 2D and 3D, they are likely to be very useful to model toxicity *in vitro*, and in the future will feature more prominently in toxicity studies. In this review, we have discussed how stem cell models as *in vitro* pre-clinical assays are rapidly evolving. These systems are sensitive to specific genome modification and the assessment of a patient's susceptibility to a drug before clinical exposure. The rapid advancement of these systems to accurately predict patient response, particularly in the liver, heart and GI systems, will greatly accelerate the advancement of drugs from their development to clinical use, as these organs express the greatest abundance of key drug-metabolising enzymes and consequently show the greatest susceptibility to ADRs.

## Abbreviations

ADR: adverse drug reactions (ADRs)
BSEP: bile salt export pump
CdCl_2_: cadmium chloride
CES: carboxylesterases
DCM: dilated cardiomyopathy
DIC: drug-induced cholestasis
EHEC: enterohaemorrhagic *Escherichia coli*
GI: gastrointestinal
HCM: hypertrophic cardiomyopathy
hESCs: human embryonic stem cells
hESC-CMs: human embryonic stem cell-derived cardiomyocytes
hFLPC-HLCs: human foetal liver progenitor cell-derived hepatocytes
HIO: human intestinal organoids
hPH: human primary hepatocytes
hSKP: human skin-derived precursors
iPSCs: induced pluripotent stem cells
iPSC-CM: IPSC-derived cardiomyocytes
iPSC-HLCs: iPSC-derived hepatocyte-like cells
LGR5: leucine-rich-repeat-containing G-protein-coupled receptor 5
LQT: long QT syndrome
ROS: reactive oxygen species
